# The Medical Police of London

**Published:** 1855-12

**Authors:** 


### THE MEDICAL POLICE OF LONDON.

By the provisions of the new Act for "the better Local Management of the Metropolis", a plan is presented by which the whole of the modern Babylon may be placed under constant scientific sanitary supervision. If properly carried out, this will in time become the leading feature of the Act, and will render London, which has long been, comparatively speaking, a healthy city, healthier than many of our small country towns noted for salubrity. It will, however, take some time for the due organisation of the important corps who will henceforth become the sanitary police of London; for, in the first place, amongst the hosts of applicants for the duties of the Sanitarian, it can scarcely fail to occur that some will be elected who are neither by taste nor by special education fully fitted for the duties implied; and, in the second place, the duties themselves have in a great degree to be learned and defined.

If, however, the proper men are found for the position, the duties will follow in train as a matter of necessity. It is in the selection of officers, therefore, that the Municipal Councils and District Boards must be most careful. To select for these important posts men who are acquainted only with the merest routine of medical practice, who know few mysteries deeper than the action of salts and senna, who perform cures with gum tragacanth, who believe that there is a specific difference between diluted tincture of cardamoms and brandy and water, and who accept the virtues of peppermint as an article of medical faith, while they steer clear of the great fact that the sun is the soul of the living world, and dub as a theorist the man who considers disease to be a unity and one individual problem in the arcana of nature—to place such men in the office of sanitary dictator will be to ignore efficiency, to dishonour science, and to degrade what might be a gracious and cheap boon, into a huge, costly, and vulgar exhibition.

The man who would represent fully the sanitary office, requires perhaps more extended acquirements than belongs to the majority of men who follow scientific occupations. He should have a knowledge of practical chemistry; of the symptoms, causes, and treatment of disease; of physiology, or the laws of life; of pathology, or the laws of morbid action; of forensic medicine, or the connexion that exists between the science of medicine and the law; of various matters of a mechanical kind, bearing on sewerage, house-building, street-cleansing, and water supply; of meteorology, or the effects of climate, weather, and atmospheric influences on the body; of the physical characters or dynamics of the atmosphere; of ventilation; of statistics of life and mortality; of the literature of epidemics, and of all sanitary improvements. Lastly, he should possess sound logical faculties, so that in dealing with facts and opinions he may neither mistake coincidences for causes, nor build up great theories on insignificant data, nor from great facts deduce absurd conclusions.

There is here, it is true, sketched out, a high standard of qualification; and it may be difficult to find any one who shall come up to it in all its fulness. But he who approaches it nearest, is most fit for the place in question; and for him who bears its measurement best, it is the duty of each municipal board to seek earnestly.

It has been observed that, if the proper men are elected for the duties of Officers of Health, London will soon have the best Council of Health that could be devised; and that the State might so organise this Council, and so place itself in connexion with it, as to obtain the advice and assistance of a truly scientific body in all national emergencies, when sanitary advice is required. This would be indeed a grand ultimatum; but we hope that, as soon as the elections have taken place, the officers elected will spontaneously organise themselves into an independent association for the advancement of sanitary science; or, as would be far better, link their labours with those of some existing scientific society. Of all societies most fitted for such an union, the Epidemiological stands pre-eminent; and we know of nothing that would add more lustre to this useful and distinguished association, than the connexion with it of a Sanitary Council, composed of the Medical Officers of Health of the metropolis.

A yearly report of the sanitary state of all London, drawn up by scientific and independent men, would in twenty years throw more light on the general causes of disease, and on the principles of prevention, than all the stray medical writings on these subjects which have appeared since the days of Hippocrates.

